# Adverse pregnancy outcomes in adolescents and young women with systemic lupus erythematosus: a national estimate

**DOI:** 10.1186/s12969-018-0242-0

**Published:** 2018-04-16

**Authors:** Nicole Ling, Erica Lawson, Emily von Scheven

**Affiliations:** University of California San Francisco, 550 16th Street, 5th Floor, Box 0632, San Francisco, CA 94143 USA

**Keywords:** Pregnancy, Systemic lupus erythematosus, Outcomes, Adolescent

## Abstract

**Background:**

Pregnant women with systemic lupus erythematosus (SLE) have increased risk of adverse outcomes including disease flare, spontaneous abortion, preeclampsia/eclampsia, premature birth and maternal death. However, pregnancy outcomes among adolescents and young women with SLE have not been well-explored. Our objective was to compare risk of adverse pregnancy outcomes in adolescents and young women with SLE to risk among peers without SLE.

**Methods:**

We studied the 2000–2011 Nationwide Inpatient Sample (NIS) of the Healthcare Cost and Utilization Project (HCUP) to estimate the prevalence of adverse pregnancy outcomes in women with SLE aged ≤ 21 years at time of delivery. Outcomes were compared to peers without SLE by using multivariate logistic regression to calculate odds ratios and risk differences. Additionally, differences in length of stay and total charges per hospitalization were described.

**Results:**

There were 8,791,391 unique pregnancies, of which 4002 occurred in young women with SLE. After adjustment for age, race, insurance type and quartile of median income based on patient ZIP code individuals with SLE had increased odds of pre-eclampsia/eclampsia (OR 3.2, 95% CI 2.3–4.6), maternal death (OR 80, 95% CI 10–604), preterm birth (OR 2.7, 95% CI 2–3.7), spontaneous abortion (OR 5.1, 95% CI 2.8–9.6), and induced abortion (OR 30, 95% CI 14–63). The increase in risk among women with SLE was greatest for preterm birth (RD 11%, 95% CI 6–16), pre-eclampsia/eclampsia (RD 9%, 95% CI 5–13), and spontaneous abortion (RD 4%, 95% CI 0.9–6). Risk difference for induced abortion was 2% with 95% CI 0.6–4, while the difference in risk for maternal death did not reach statistical significance (RD 0.4, 95% CI -0.4-1).

**Conclusions:**

Adolescents and young women with SLE experience increased risk of adverse, pregnancy-specific outcomes as compared to their peers, including pre-eclampsia/eclampsia, maternal death, preterm birth, spontaneous abortion, and induced abortion. Additionally, length of stay and total charges for hospitalization are increased.

**Electronic supplementary material:**

The online version of this article (10.1186/s12969-018-0242-0) contains supplementary material, which is available to authorized users.

## Background

Systemic Lupus Erythematosus (SLE) is a complex autoimmune condition that affects adolescents and young women of childbearing age. When women with SLE become pregnant, numerous manifestations of SLE and its associated treatment can affect both maternal and fetal outcomes. Prevalence of adverse pregnancy outcomes in the SLE adult population have been well documented, including estimates from several single-center cohort studies from across the world [1–10]. Comparative studies using national registries have reported increased risk of preterm labor, fetal growth restriction, hypertensive disorders including pre-eclampsia and eclampsia, maternal death, and fetal death in SLE [11–13]. Women with SLE are also at increased risk of infectious, hematologic and thrombotic maternal complications. Risk factors associated with poor pregnancy outcomes among women with SLE include active disease, renal involvement, presence of anti-phospholipid antibodies, use of high doses of glucocorticoids, and fewer years of education [14–18].

Young women and adolescents are at increased risk of unintended pregnancy as compared to older women [19]. Sexual behavior of adolescents with chronic health conditions has been shown to be no different than that of their peers without chronic conditions, putting young women with SLE at the same risk of unintended pregnancy [20]. Furthermore, there is increased incidence of renal involvement among adolescents with SLE as compared to adults with SLE, which may put adolescents at greater risk of poor obstetric outcomes if they become pregnant [21].

In spite of these increased risks, pregnancy incidence and outcomes among adolescents and young women with SLE remain understudied. In the last ten years, only one study has attempted to capture pregnancy risk in this population [22]. Prevalence of maternal and fetal outcomes was described in a multi-center, Brazilian cohort of 24 pregnancies in 23 patients with pediatric-onset SLE, aged 14–21.7 years. Using multivariate modeling, inadvertent intravenous cyclophosphamide use at the start of pregnancy was the only independent risk factor for fetal loss. However, the sample size for the study was small and comparative measures of risk between those with and without SLE were not explored.

In the current study, we used a national dataset to estimate risk of adverse, pregnancy-specific outcomes among adolescents and young women with SLE, focusing on the age range typically cared for by pediatric rheumatologists.

## Methods

### Data source

We studied the 2000–2011 Nationwide Inpatient Sample (NIS), part of the Agency for Healthcare Research and Quality’s Healthcare Cost and Utilization Project (HCUP) [23]. The NIS is the largest all-payer, inpatient care database in the United States, containing demographic and clinical information obtained from hospital discharge abstracts. Available yearly since 1988, each year of the NIS contains data on approximately 8 million hospital stays from about 1000 hospitals, sampled to approximate a 20% stratified sample of U.S. community hospitals. Hospitals are divided into strata based on U.S. region, urban/rural location, teaching status, bed size and ownership. Sampling probabilities are proportional to the number of hospitals in each stratum. No unique patient identifiers are contained in the NIS, as the unit of analysis is individual hospitalization, rather than patient. Information available for each hospitalization includes general hospital characteristics as well as patient information such as age, gender, race, quartile of median income based on patient ZIP code, and discharge diagnoses/procedure codes from the International Classification of Diseases, Ninth Revision, Clinical Modification (ICD-9-CM). Discharge diagnoses include one primary and up to 24 secondary discharge diagnoses as well as up to 15 procedural codes.

### Study population

We identified unique pregnancies among individuals with and without SLE age 14–21 years. We chose to include individuals up to age 21 because previous survey data demonstrates that nearly half of pediatric rheumatologists follow patients until age 21 or older [24]. Hospitalizations of patients with SLE were identified with ICD-9 code 710.0. The youngest, pregnant patient with SLE was 14 years of age at time of delivery, so analysis was restricted to hospitalizations of pregnant women aged 14 or older. Hospitalizations of pregnant women aged 14–21 were included. Unique pregnancies were identified for analysis by including only those hospitalizations in which ICD-9 diagnosis or procedural codes specified delivery, live born, spontaneous or induced abortion, ectopic pregnancy or intrauterine death. ICD-9 codes for these conditions were identified using HCUP Clinical Classifications Software (CCS) and incorporated for inclusion/exclusion using ICD9x for STATA 13.0 software [25–27].

### Measures

The primary outcome was pregnancy-specific adverse maternal or fetal outcome. Maternal outcomes included pre-eclampsia/eclampsia and death. Fetal outcomes included preterm birth (early onset of delivery, immaturity or birth before 37 weeks gestation), spontaneous abortion (unspecified abortion or intrauterine death), induced abortion, and ectopic pregnancy. Data on other fetal outcomes such as weight, growth and congenital anomalies were not reliably available from maternal records, and therefore were not included. ICD-9 codes for each of the above variables are available in tables provided in the Additional file 1. Additionally, length of stay and total charges per hospital stay were included as secondary outcome variables. Predictor variables included age, race, socioeconomic status (SES) and insurance statues. Race was categorized as White, Black, Hispanic, Asian or Pacific Islander, Native American, or Other. SES was categorized according to quartile of median household income based on patient ZIP code. Insurance status was defined as primary expected payor.

### Statistical analysis

Sample weights were applied to account for stratified sampling design and multiple years of data, generating nationally representative estimates. Survey data commands were used for all descriptive and inferential analyses. Unadjusted linear regression was used to compare age, length of stay and total charges in those with and without SLE. Chi squared analyses were used to compare weighted cell frequencies and their proportions. Adjusted logistic regression was used to compute odds ratios and 95% confidence intervals for the primary outcome of pregnancy-specific adverse maternal and fetal outcomes among women aged 14–21 with and without SLE. Marginal estimates were calculated to obtain risk difference for these outcomes. Both the odds ratio (OR) and risk difference (RD) calculations were performed after adjusting for age, race, SES and insurance. There was missing data for some observations of race, as some hospitals and HCUP State Partners do not supply this information. Therefore, to account for missing data, inverse probability weights were generated using logistic regression to model for race “missingness” and then applied prior to calculation of adjusted risk estimates [23, 28]. The logistic regression model for race “missingness” included patient age, number of chronic diseases, NIS stratum, quartile of median income based on patient ZIP code, SLE status, and all maternal pregnancy outcome variables. Unstabilized weights were used due to the lack of variation in weights. Descriptive statistics were used to compare secondary outcomes, length of stay and total charges per hospital stay, among women with and without SLE.

All statistical analyses were performed using STATA software, version 13.0 (StataCorp, College Station, TX.)

## Results

### Obstetric hospitalizations

From 2000 to 2011, there were 8,791,391 hospitalizations for unique pregnancies among young women age 14–21 (Table 1). Among these hospitalizations, 4002 included a discharge diagnosis of SLE. The mean age was 19.4 years in the SLE group and 19.0 in the non-SLE group (*p* < 0.001). Distribution of race across SLE and non-SLE differed (p < 0.001). The non-SLE group included more Caucasians (43% vs. 35%), while the SLE group included more African Americans (34% vs. 21%). There was no difference in socioeconomic status between SLE and non-SLE groups, as represented by median household income quartile based on patient zip code. A difference in insurance type between the two groups was detected. Those with SLE had a slightly increased prevalence of private insurance (26% vs. 24%) and a slightly lower prevalence of Medicaid (67% vs. 69%) and self-pay (2.2% vs. 3.8%) when compared to those with SLE (*p* < 0.001).

**Table 1:** Demographics of pregnant, hospitalized, young women with and without SLE, age 14–21

Demographic	SLE (*n* = 4002)	Non-SLE (*n* = 8,787,389)	*P*
	N (%) or Mean (± SD)		
Age at delivery, years	19.4 ± 0.05	19.0 ± 0.01	< 0.001
Race (*n* = 6,646,192)			< 0.001
White	1107 (35)	2,837,585 (43)	
Black	1070 (34)	1,402,261 (21)	
Hispanic	828 (26)	1,937,211 (29)	
Asian or Pacific Islander	43 (1.3)	114,299 (1.7)	
Native American	39 (1.2)	62,090 (0.93)	
Other	91 (2.9)	289,569 (4.4)	
Socioeconomic Status^a^(n = 8,622,189)			0.42
1	1273 (32)	2,738,962 (32)	
2	1134 (29)	2,581,298 (30)	
3	853 (22)	1,995,447 (23)	
4	665 (17)	1,302,558 (15)	
Insurance Type (n = 8,774,388)			< 0.001
Medicare	43 (1.1)	29,987 (0.3)	
Medicaid	2666 (67)	6,082,943 (69)	
Private, including HMO	1048 (26)	2,070,621 (24)	
Self-pay	89 (2.2)	329,328 (3.8)	
No charge	9 (0.2)	22,538 (0.3)	
Other	138 (3.4)	234,979 (2.7)	

^a^As represented by median household income quartiles for patient’s ZIP code: 1 = lowest income, 4 = highest income

### Adverse pregnancy outcomes

Prevalence of pre-eclampsia and eclampsia, maternal death, preterm birth, spontaneous abortion, and induced abortion were increased in those with SLE compared to those without SLE in unadjusted analysis (Table 2). After adjusting for age, race, insurance type, and quartile of median household income based on patient ZIP code, SLE was associated with increased odds of pre-eclampsia/eclampsia (OR 3.2, 95% CI 2.3–4.6), maternal death (OR 80, 95% CI 10–604), preterm birth (OR 2.7, 95% CI 2–3.7), spontaneous abortion (OR 5.1, 95% CI 2.8–9.6), and induced abortion (OR 30, 95% CI 14–63). Ectopic pregnancies were rare in this population and differences across those with and without SLE did not reach statistical significance.

**Table 2:** Adverse pregnancy outcomes of young women age 14–21 with and without SLE

Outcome	SLE (n = 4002)	Non-SLE (n = 8,787,389)	OR (95%CI) ^a^	Risk Difference % (95%CI)^a^
	N (%)			
Maternal				
Pre-eclampsia or eclampsia	636 (16)	401,549 (4.6)	3.2 (2.3–4.6)	9 (5–13)
Death	15 (0.38)	465 (0.005)	80 (10–604)	0.4 (−0.4–1)
Fetal				
Preterm Birth	818 (20)	707,921 (8.1)	2.7 (2–3.7)	11 (6–16)
Spontaneous abortion or Intrauterine death	221 (5.5)	100,130 (1.1)	5.1 (2.8–9.6)	4 (0.9–6)
Induced abortion	87 (2.2)	11,175 (0.13)	30 (14–63)	2 (0.6–4)
Ectopic pregnancy	≤10 (≤0.24)^b^	29,095 (0.3)	0.8 (0.12–6)	− 0.1 (− 0.7–0.6)

^a^Adjusted for age, race, insurance type, and quartile of median income based on patient ZIP code

^b^Unable to report cell sizes less than or equal to 10, per HCUP data use regulations

The increase in risk among women with SLE was greatest for preterm birth (RD 11%, 95% CI 6–16), pre-eclampsia/eclampsia (RD 9%, 95% CI 5–13), and spontaneous abortion (RD 4%, 95% CI 0.9–6). Risk difference for induced abortion was 2% with 95% CI 0.6–4, while the difference in risk for maternal death did not reach statistical significance (RD 0.4, 95% CI -0.4-1).

### Healthcare utilization

Length of stay and total charges per hospitalization were compared between the SLE and non-SLE group. Median length of stay for those with SLE was 3 days (interquartile range (IQR) 2–4 days) compared to 2 days (IQR 1–3 days) in those without SLE (*p* < 0.001). Median total charges for hospitalization for those with SLE was $11,146 (IQR $6973–$19,187) compared to $7198 (IQR $4824–$11,172) for those without SLE (p < 0.001).

## Discussion

This study demonstrated an increased risk of adverse pregnancy-related outcomes among adolescents and young women with SLE as compared to their peers without SLE, utilizing a large nationwide dataset. Compared to those without SLE, pregnant young women with SLE were at increased risk for pre-eclampsia/eclampsia, maternal death, spontaneous and induced abortion, and preterm birth. Prior studies have not focused on the population typically cared for by pediatric rheumatologists, in spite of the fact that pregnancy occurs frequently among adolescents, including those with SLE [2, 3].

The increased risk of adverse, pregnancy-specific outcomes among adolescents and young women with SLE is consistent with previous findings in the literature among adult women with SLE. Studies using national registries have reported increased risk of preterm labor (OR 2.4–4.0), fetal growth restriction (OR 2.6–5.0), hypertensive disorders including pre-eclampsia and eclampsia (OR 3–4.4), maternal death (OR 17.8), and fetal death (OR 2.4–7) [11–13] Estimates of risk of maternal death in those aged 21 or less are slightly higher than previously published work looking at adult populations. However, it should be noted that this outcome was still a rare occurrence and our estimates included a wide confidence interval. Risk differences were calculated to further explore the risk of maternal death. Risk difference calculations take into account the prevalence of each outcome in the study population. Though maternal death and induced abortion had the highest odds ratios, these outcomes had the lowest differences in risk, and the risk difference for maternal death was not significant. The findings of increased adverse pregnancy outcomes in those with SLE are further strengthened by detected differences in median length of stay and total charges for hospitalization.

Because of the sampling methodology used in the study design of the NIS database, our results are generalizable to hospitals included in the sampling frame. In 2011, the NIS captured over 97% of the U.S. population hospital discharges from 46 states. Therefore, our results are generalizable to nearly the entire U.S. population.

Use of this large, nationally-representative dataset has inherent limitations. First, classification of SLE and adverse maternal pregnancy outcomes were based on ICD-9 discharge diagnosis codes. These discharge diagnosis codes are subject to coding error and misclassification. We were unable to validate the sensitivity or specificity of coding in this study, however, previous research has estimated the positive predictive value (PPV) of ICD-9 billing codes for SLE and SLE nephritis in Medicaid patients. These authors studied several algorithms to attempt to capture patients with SLE and SLE nephritis. Their algorithms included three or more ICD-9 codes for SLE (710.0) and either/both of the following: three or some renal ICD-9 codes and 3 or more nephrology visits. Using these algorithms combined with chart review, PPV for SLE ranged from 89 to 92%) [29]. As mentioned previously, there are no unique patient identifiers in the NIS database, therefore inclusion of repeated ICD-9 codes to define SLE in this study was not possible. Another study based in Canada calculated sensitivity, specificity, PPV and negative predictive values (NPV) of ICD-9 codes. They reported a sensitivity of 89.8, specificity of 98.7, PPV of 99.9, and NPV of 0.64 for detecting rheumatic disease with ICD-9 coding in their database [30]. Of note, misclassification of SLE in this study would decrease our ability to detect differences between the two groups.

Second, we are limited by the amount of information provided by hospital discharge abstracts. We were unable to examine the impact of SLE related covariates such as disease duration, damage, and activity. Furthermore, effects of medications and behavioral characteristics of patients (adherence, access to prenatal care) on adverse pregnancy-specific outcomes could not be explored due to lack of medical information in the dataset. Third, we were unable to link maternal hospital discharge records to corresponding fetal hospital discharge records, limiting our ability to explore fetal-specific outcomes, including length of stay and total charges for hospitalization. Finally, the NIS contains only data from hospitalizations. Deliveries and abortions that did not occur in the hospital setting are not accounted for in this analysis, which may bias estimates if those with SLE are more likely to present to the hospital for delivery and abortion as compared to women without SLE. In spite of these limitations, the size of this dataset enabled us to estimate risk for rare events of great interest to patients and clinicians, specifically adverse pregnancy-specific outcomes among young women with SLE, that might otherwise not be possible.

## Conclusions

These results are the first to quantify risk of adverse, pregnancy-specific outcomes in adolescents and young women with SLE on a national scale. The increase in risk among women with SLE was greatest for preterm birth, pre-eclampsia/eclampsia, and spontaneous abortion. This work provides practitioners with tangible estimates of risk for young women and adolescents that can be leveraged when discussing reproductive health and when counseling adolescents who become pregnant. Further studies to identify specific disease and treatment related risk factors in the adolescent and young women population are needed.

## Additional file

Additional file 1: **Table S1a.** ICD9 diagnosis and procedure codes used to identify hospitalizations for unique pregnancies. **Table S1b.** ICD9 diagnosis codes used to identify pregnancy outcomes. (PDF 50 kb)

## Abbreviations

CI: confidence interval
HCUP: Healthcare Cost and Utilization Project
ICD-9-CM: International Classification of Diseases, Ninth Revision, Clinical Modification
NIS: Nationwide Inpatient Sample
NPV: negative predictive value
OR: odds ratio
PPV: positive predictive value
RD: risk difference
SLE: systemic lupus erythematosus
U.S.: United States
ZIP code: zone improvement plan code
