# Supplementary material for: Adverse pregnancy outcomes in adolescents and young women with systemic lupus erythematosus: a national estimate
Source: Pediatr Rheumatol Online J. 2018 Apr 16;16:26. doi: 10.1186/s12969-018-0242-0 (PMC5902884; doi:10.1186/s12969-018-0242-0)
Supplement: Supplementary file 1 — Table S1a. ICD9 diagnosis and procedure codes used to identify hospitalizations for unique pregnancies. Table S1b. ICD9 diagnosis codes used to identify pregnancy outcomes. (PDF 50 kb) [file 12969_2018_242_MOESM1_ESM.pdf]

## Table S1a: ICD9 diagnosis and procedure codes used to identify hospitalizations for unique pregnancies

| | | Multilevel CCS code | ICD9 Code |
|---|---|---|---|
| Diagnosis codes: | Forceps delivery | 11.5.3 | 669.50 669.51 |
| | Normal delivery | 11.7.1 | 650 |
| | Outcome of delivery | 11.7.3 | V22.0 V22.1 V22.2 V24.0 V24.1 V24.2 V27.0 V27.1 V27.2 V27.3 V27.4 V27.5 V27.6 V27.7 V27.9 V72.4 V72.42 |
| | Liveborn | 15.1 | 765.20 765.29 V30.0 V30.00 V30.01 V30.1 V30.2 V31.0 V31.00 V31.01 V31.1 V31.2 V32.0 V32.00 V32.01 V32.1 V32.2 V33.0 V33.00 V33.01 V33.1 V33.2 V34.0 V34.00 V34.01 V34.1 V34.2 V35.0 V35.00 V35.01 V35.1 V35.2 V36.0 V36.00 V36.01 V36.1 V36.2 V37.0 V37.00 V37.01 V37.1 V37.2 V39.0 V39.00 V39.01 V39.1 V39.2 |
| | Spontaneous abortion | 11.2.1 | 634.00 634.01 634.02 634.10 634.11 634.12 634.20 634.21 634.22 634.30 634.31 634.32 634.40 634.41 634.42 634.50 634.51 634.52 634.60 634.61 634.62 634.70 634.71 634.72 634.80 634.81 634.82 634.90 634.91 634.92 |
| | Induced abortion | 11.2.2 | 635.00 635.01 635.02 635.10 635.11 635.12 635.20 635.21 635.22 635.30 635.31 635.32 635.40 635.41 635.42 635.50 635.51 635.52 635.60 635.61 635.62 635.70 635.71 635.72 635.80 635.81 635.82 635.90 635.91 635.92 636.00 636.01 636.02 636.10 636.11 636.12 636.20 636.21 636.22 636.30 636.31 636.32 636.40 636.41 636.42 636.50 636.51 636.52 636.60 636.61 636.62 636.70 636.71 636.72 636.80 636.81 636.82 636.90 636.91 636.92 637.00 637.01 637.02 637.10 637.11 637.12 637.20 637.21 637.22 637.30 637.31 637.32 637.40 637.41 637.42 637.50 637.51 637.52 637.60 637.61 637.62 637.70 637.71 637.72 637.80 637.81 637.82 637.90 637.91 637.92 638.0 |
| | Ectopic Pregnancy | 11.3.1 | 633.0 633.00 633.01 633.1 633.10 633.11 633.2 633.20 633.21 633.8 633.80 633.81 633.9 633.90 633.91 |
| | Intrauterine death | 11.6.5 | 656.40 656.41 656.43 |
| Procedure codes: | Cesarean section | 13.2 | 74.0 74.1 74.2 74.4 74.99 |
| | Forceps, vacuum and breech delivery | | 72.1 72.71 72.79 72.00 72.21 72.29 72.31 72.39 72.4 72.51 72.52 72.53 72.54 72.6 72.8 72.9 73.3 |
| | Abortion | 12.7 | 69.01 69.51 74.91 75.0 |

## Table S1b: ICD9 diagnosis codes used to identify pregnancy outcomes

| Outcomes | Multilevel CCS code | ICD9 Code |
|---|---|---|
| Preeclampsia and eclampsia | 11.3.3.1 | 642.40 642.41 642.42 642.43 642.44 642.50 642.51 642.52 642.53 642.54 642.60 642.61 642.62 642.63 642.64 642.70 642.71 642.72 642.73 642.74 |
| Preterm Birth | 11.3.4.2 | 644.20 644.21 765.0 765.00 765.01 765.02 765.03 765.04 765.05 765.06 765.07 765.08 765.09 765.1 765.10 765.11 765.12 765.13 765.14 765.15 765.16 765.17 765.18 765.19 765.21 765.22 765.23 765.24 765.25 765.26 765.27 765.28 |
| Ectopic Pregnancy | 11.3.1 | 633.00 633.01 633.1 633.10 633.11 633.2 633.20 633.21 633.8 633.80 633.81 633.9 633.90 633.91 |
| Induced abortion | 11.2.2 | 635.00 635.01 635.02 635.10 635.11 635.12 635.20 635.21 635.22 635.30 635.31 635.32 635.40 635.41 635.42 635.50 635.51 635.52 635.60 635.61 635.62 635.70 635.71 635.72 635.80 635.81 635.82 635.90 635.91 635.92 636.00 636.01 636.02 636.10 636.11 636.12 636.20 636.21 636.22 636.30 636.31 636.32 636.40 636.41 636.42 636.50 636.51 636.52 636.60 636.61 636.62 636.70 636.71 636.72 636.80 636.81 636.82 636.90 636.91 636.92 |
| Spontaneous abortion | | 634.00 634.01 634.02 634.10 634.11 634.12 634.20 634.21 634.22 634.30 634.31 634.32 634.40 634.41 634.42 634.50 634.51 634.52 634.60 634.61 634.62 634.70 634.71 634.72 634.80 634.81 634.82 634.90 634.91 634.92 637.00 637.01 637.02 637.10 637.11 637.12 637.20 637.21 637.22 637.30 637.31 637.32 637.40 637.41 637.42 637.50 637.51 637.52 637.60 637.61 637.62 637.70 637.71 637.72 637.80 637.81 637.82 637.90 637.91 637.92 656.40 656.41 656.43 |
